# Street Collections for Charities

**Published:** 1895-09-14

**Authors:** 


### Street Collections for Charities.

Mr. C. S. Loch, of the Charity Organisation Society, strongly urges, in the *Times*, the fuller consideration of the question of street begging on behalf of all kinds of charities. He, not unnaturally, seeks to direct attention to what he considers the better methods which "organised charity" has put in operation. But street begging for charities seems to us to be sufficiently condemned on its own demerits. It is a most undignified proceeding, and quite contrary to the genius of the English character, which hates beggars and begging in all their forms; it is a most costly and laborious method of producing very poor results; and it can hardly fail to be very injurious to the work of charity as a whole, in that it offers such easy facilities for salving the conscience. But the greatest of all the objections to this form of begging seems to us to be its liability to abuse. We ourselves have noticed, on more than one occasion, persons dressed like young ladies, with collecting-boxes in their hands, soliciting contributions for objects of which we had never before heard so much as even the names. In point of fact, if one institution is allowed to beg in the streets, there is no reason in law or logic to prevent ten thousand others doing the same. The whole question should be fully considered by the County Council, which might usefully follow the lead of the best administered provincial cities, which have, we understand, abolished the nuisance by bye-law.

